# Fate of hydrogen sulphide on refractory oxide nanoparticles *via* foundation model-accelerated atomistic modelling and *in situ* spectroscopy

**DOI:** 10.1039/d6nr02993k

**Published:** 2026-11-02

**Authors:** Eric Mates-Torres, Lorenzo Mino, Guillermo Escolano-Casado, Nadia Balucani, Piero Ugliengo, Albert Rimola

**Affiliations:** a Departament de Química, Universitat Autònoma de Barcelona 08193 Bellaterra Catalonia Spain eric.mates@uab.cat albert.rimola@uab.cat; b Dipartimento di Chimica and Nanomaterials for Industry and Sustainability (NIS) Centre, Università degli Studi di Torino via P. Giuria 7 I-10125 Torino Italy lorenzo.mino@unito.it; c Dipartimento di Chimica, Biologia e Biotecnologie, Università degli Studi di Perugia Via Elce di Sotto 8 I-06123 Perugia Italy; d Accademia delle Scienze di Torino Via Maria Vittoria 3 I-10123 Torino Italy

## Abstract

Understanding the intricate interactions between hydrogen sulphide (H_2_S) and complex metal oxides is of paramount interest for both advancing industrial catalysis, such as H_2_S conversion to H_2_, and fundamental perspectives, such as unravelling the role of refractory interstellar dust in the sulphur depletion problem or in prebiotic catalysis. In both settings, the chemistry is governed by the high-surface-area, structurally heterogeneous terminations that dominate at the nanoscale, including facets of differing stability, steps, and point defects; however, the vast configurational space of these chemically diverse and reactive interfaces makes exhaustive first-principles sampling prohibitively expensive. In this work, we address this challenge by integrating unbiased, automated high-throughput adsorption sampling with readily available atomistic foundation models in a hierarchical screen-and-refine workflow. We systematically investigate H_2_S adsorption across three prototypical refractory oxide materials: amorphous silica, forsterite (Mg_2_SiO_4_), and pristine and defective MgO. A state-of-the-art foundation model was used to explore, without chemical bias, the configurational space of H_2_S–surface interactions, revealing a broad range of surface phenomena, transitioning from non-covalent, hydrogen-bonded physisorption on hydroxylated silica to dissociative adsorption on Mg_2_SiO_4_ facets and highly reactive Schottky divacancy pairs in MgO. The resulting atomistic datasets are assessed against experimental *in situ* infrared (IR) spectroscopy. Our multiconfigurational compound IR spectra, computed at the density functional theory level for a set of machine-learning potential refined minima, reproduce the position, ordering and qualitative shape of the experimental features, including the complex low-frequency tails and the progressive red-shifting of characteristic H–S and O–H stretching modes across the three materials. This combined theoretical and experimental framework establishes a transferable methodology for the study of refractory nanostructured surfaces in Earth and astrochemical settings, and provides a pipeline for the rational design of next-generation catalysts for sustainable processes and green chemistry applications.

## Abstract 2

A foundation machine-learned potential screens the adsorption of hydrogen sulphide on silica, forsterite and MgO, and DFT refines every candidate. The DFT-simulated IR spectra reproduce the order of the H–S stretching bands measured *in situ*.

## Graphical abstract

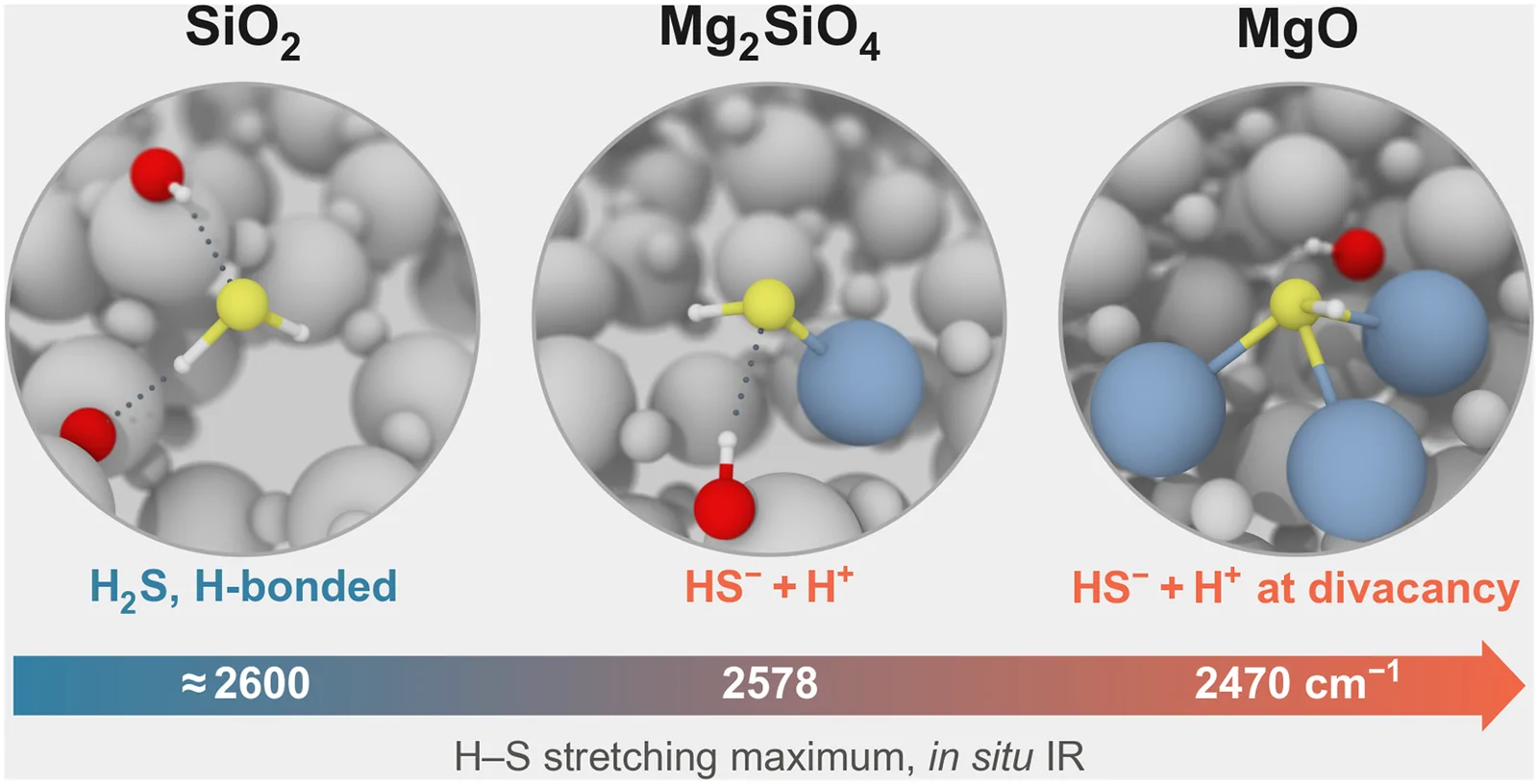

## Introduction

Hydrogen sulphide (H_2_S) plays a central role in heterogeneous catalysis and in the chemistry of natural solid–gas interfaces. In the industrial context, H_2_S is ubiquitously present in crude oil and natural gas (commonly referred to as sour gas) and is generated as a major byproduct during their processing. Owing to its high toxicity and chemical reactivity, the direct release of H_2_S into the atmosphere is not viable, prompting the development of large-scale H_2_S retrieval and conversion strategies. Among these, the Claus process remains the most widely implemented industrial route, converting H_2_S into elemental sulphur and water over oxide-based catalysts such as Al_2_O_3_ and TiO_2_.^1^ During the catalytic stages of the Claus process, H_2_S has been shown to adsorb dissociatively on the catalyst surface and subsequently react with incoming SO_2_. In this process, the accumulation of sulphur species on the catalyst surface ultimately leads to surface site poisoning and catalyst deactivation. Similar sulphur-induced deactivation phenomena are also encountered in the synthesis of value-added chemical feedstocks from syngas, where even trace amounts of H_2_S hinder the activity of Fischer–Tropsch catalysts.^2^ For this reason, alternative pathways for H_2_S capture and conversion have been proposed, particularly the direct decomposition of H_2_S on Earth-abundant catalytic materials to produce H_2_, shifting H_2_S from an environmental liability to a potential feedstock for hydrogen generation relevant to decarbonization strategies.^3^ For this reason, substantial experimental and theoretical efforts have been devoted to understanding how H_2_S adsorbs and dissociates on a wide range of catalyst classes, including metals,^2,4–6^ metal oxides,^7–9^ metal sulphides,^10–14^ and single-atom catalysts.^15^

Beyond industrial catalysis, H_2_S is of fundamental importance in astrochemical environments, since it has long been proposed as a major reservoir of sulphur in the interstellar medium (ISM); however, its observed gas-phase abundance falls far below expectations based on cosmic elemental abundances. One leading hypothesis attributes the missing sulphur to molecular sequestration on the surfaces of interstellar dust grains and their parent bodies. In this context, adsorption and potential dissociation of H_2_S on refractory minerals, which constitute the solid component of interstellar dust, have been recently investigated using atomistic simulations. Owing to the Lewis acid–base character of their surface sites, theoretical studies have shown that these minerals are capable of binding and activating a wide range of molecules, from simple molecules to relevant biomolecules detected in meteoritic and cometary samples.^16–22^ Notably, previous theoretical studies have shown that H_2_S can dissociate on olivine surfaces and nanoclusters, particularly on high-energy terminations, potentially leading to sulphur incorporation into the mineral lattice and accounting for its systematic non-detection in astronomical observations.^23^

In both catalytic and astrochemical settings, an accurate atomistic description of the molecule–surface interface is therefore essential. However, a key limitation of conventional adsorption studies lies in the difficulty of exhaustively sampling the vast configurational space associated with molecule–surface interactions. This becomes particularly challenging for complex nanostructured oxide materials, such as those relevant to astrochemical settings, which exhibit intricate surface morphologies, multiple coordination environments, and chemically diverse reactive sites. To address this issue, automated adsorption workflows based on grid sampling and semi-empirical methods have been developed and successfully applied to study the adsorption of prebiotic reaction precursors on mineral surfaces.^20,24,25^ While these approaches enable efficient exploration of adsorption landscapes, their limited chemical accuracy restricts their ability to reliably describe processes beyond chemisorption, such as bond activation and dissociation, which are key aspects of H_2_S surface chemistry.

In this work, we overcome these limitations by combining automated high-throughput adsorption sampling with state-of-the-art machine-learned foundation models capable of rapidly describing physisorption, chemisorption, and bond breaking in chemical interfaces.^26^ The considered machine-learned interatomic potentials (MLPs) approximate the potential energy surface by regression on first-principles data, reproducing DFT energies and forces at a cost several orders of magnitude lower and with a scaling that allows the relaxation of thousands of candidate configurations at a fraction of the DFT cost.^27,28^ Recent foundation MLPs extend this idea by training a single model on broad inorganic datasets spanning most of the periodic table, so that they can be applied as-is to a new material without system-specific fitting. Their training data are, however, overwhelmingly composed of bulk crystals, which place undercoordinated surface sites, point defects, and bond-breaking events outside the domain in which they were fitted; in these regimes, the accuracy of a foundation model is not guaranteed, and must be evaluated for the systems of interest. This motivates the hierarchical strategy adopted here: the foundation model is used to locate candidate adsorption motifs across a configurational space too large for DFT, while every structure that survives screening is re-optimised at the DFT level, so that all reported energetics and vibrational properties rest on first-principles calculations.

We investigate the adsorption of H_2_S on three prototypical nanostructured oxide systems with markedly different chemical behaviours toward adsorbates: MgO, hydroxylated amorphous silica (hereafter referred to as silica), and forsterite (Mg_2_SiO_4_), the magnesium end-member of olivines. Additionally, we consider MgO(100) surfaces containing Schottky-type point defects, which involve the removal of stoichiometric Mg–O pairs to maintain surface charge neutrality and better represent the reactive nature of the experimental interface.^29^ Two distinct geometric configurations of this single Schottky defect are considered: MgO(100) with a nearest-neighbour (NN) defect, where the vacant Mg and O sites are adjacent, and a next-nearest-neighbour (NNN) defect, where the vacancies are separated by one lattice site. These materials are widely employed as model oxides and catalyst supports and are also among the most abundant refractory solids forming the parent bodies of interstellar dust. Together, the studied materials span a broad spectrum of surface reactivity, encompassing non-covalent physisorption, intact chemisorption, and dissociative adsorption. The computational results are then directly validated by comparison with the results from a series of *in situ* IR spectroscopy experiments performed on materials outgassed at high temperature to obtain surface conditions directly comparable to the computational models. The experiments are conducted at cryogenic temperatures (150 K) to enhance the interaction of H_2_S even with the weakest adsorption sites.

The objective of this work is therefore twofold. First, we aim to assess whether a readily available foundation MLP, used without system-specific fitting, can explore without chemical bias the adsorption landscape of H_2_S on structurally complex oxide surfaces and locate local minima, which are subsequently refined at the DFT level. Second, we aim to determine the fate of H_2_S on amorphous silica, Mg_2_SiO_4_ and pristine and defective MgO, namely whether it physisorbs, adsorbs molecularly or dissociates on each surface, and to validate the resulting atomistic picture by comparing the multiconfigurational IR spectra computed at the DFT level with the *in situ* IR spectra recorded at 150 K.

## Results and discussion

### Assessment of foundation models for adsorbate discovery

To assess the capability of MLPs to accurately simulate H_2_S adsorption, a reference dataset was constructed using periodic density functional theory (DFT) calculations at the PBE-D2*(*N*) level. The Mg_2_SiO_4_(001) surface was selected for this initial phase due to its ability to chemisorb H_2_S in both molecular and dissociative forms in preliminary calculations, providing a broad representation of the chemical nature of H_2_S on all studied interfaces. Five atomistic foundation models were benchmarked against a common set of 43 initial geometries, which converge to 16 distinct DFT adsorption structures (8 molecular and 8 dissociative), following the structural and energetic procedure detailed in the Methods section. Of the five models, MACE's MPA re-finds every one of the 16 reference structures within a local RMSD of 0.4 Å, including all four of the most strongly bound configurations (Δ*E*_ads_ < −60 kcal mol^−1^), which are the dissociated species expected to dominate the simulated vibrational spectra; in contrast, UMA-OC20 and OMAT24 recover only two of these four (Fig. 1a).

**Fig. 1:**
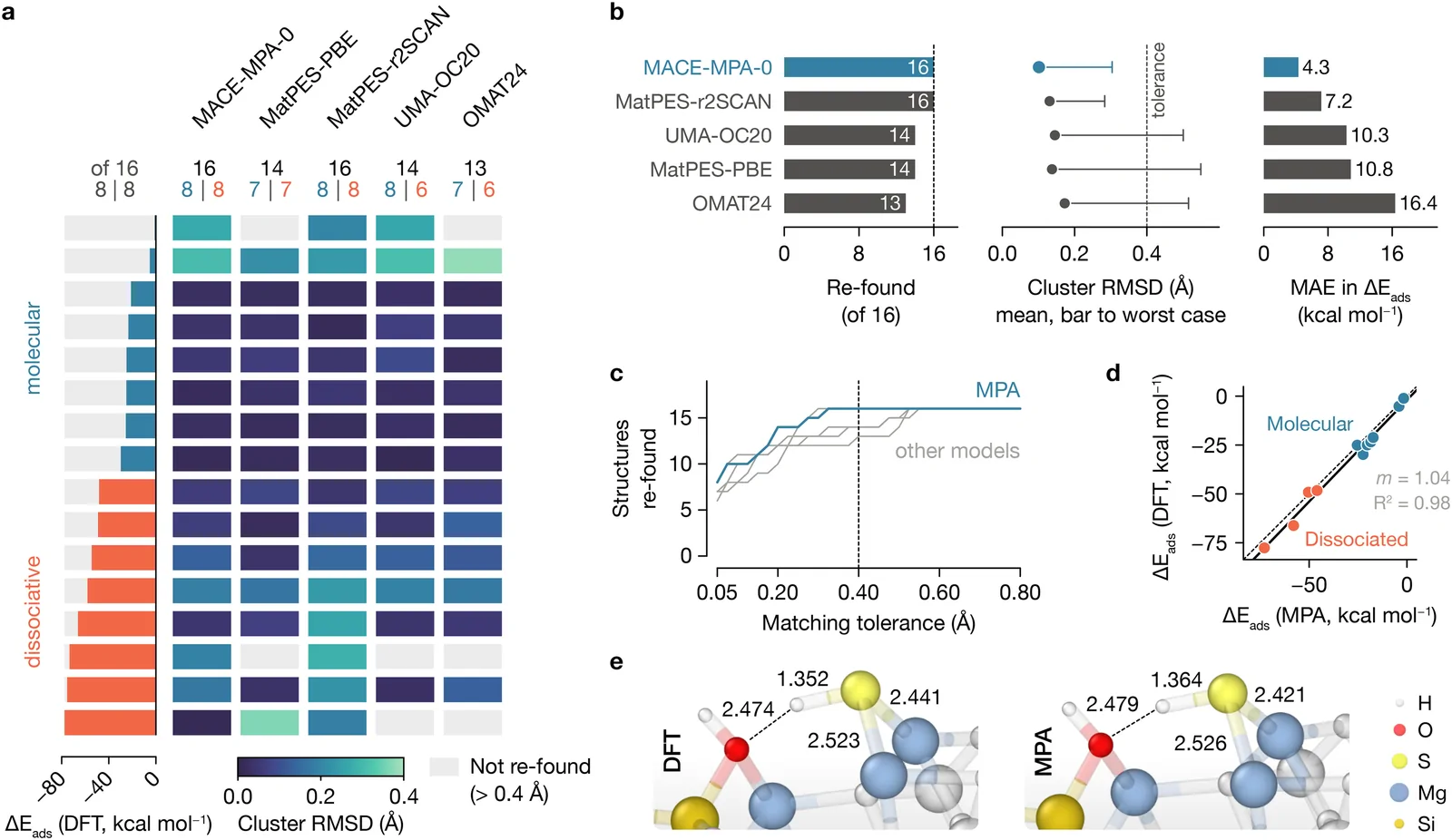
Performance of five foundation MLPs in reproducing the PBE-D2*(*N*) adsorption landscape of H_2_S on Mg_2_SiO_4_(001), from a common set of 43 initial adsorption geometry guesses. (a) Each of the 16 unique DFT adsorption structures (ordered by adsorption energies, with the left-hand bars showing different adsorption regimes: blue = molecular and orange = dissociative) against each model. Cells are shaded by the RMSD of the local environment (*cluster*) of the MLP compared to its DFT counterpart: the darker cells depict adsorptions where the MLP more accurately converged to the DFT minima. The light grey cells depict DFT structures that the corresponding MLP failed to re-find within an RMSD of 0.4 Å. The column headers show the total re-found DFT structures (out of 16) and how many of those are molecular and dissociative (in blue and orange, respectively). (b) Structures re-found, cluster RMSD (mean shown as a point, with bars extending to the worst-case value), and MAE in the adsorption energy, evaluated on the same 16 DFT structures for each MLP. (c) Structures re-found as a function of the matching RMSD tolerance. (d) Adsorption energies from the MPA MLP against PBE-D2*(*N*) for the matched pairs, with the regression slope and *R*^2^. (e) Side-view representation of the lowest-adsorption energy configuration match found by means of the automated sampling at the PBE-D2*(*N*) level (left-hand panel) and through the MPA foundation model (right-hand panel). Relevant interatomic distances are displayed in Å. Adsorbate and surface connecting atoms are displayed in colour, while all other surface atoms are whitened out for visual clarity.

Given that a recovery count alone does not allow us to discriminate between MPA and MatPES-r2SCAN, which also re-finds all DFT minima, Fig. 1b reports three measures on the same reference adsorption dataset: MPA leads on all of them, with a mean cluster RMSD of 0.101 Å against 0.131 Å and a mean absolute error (MAE) in the adsorption energy of 4.3 against 7.2 kcal mol^−1^. This ranking is insensitive to the matching tolerance over the range of 0.05–0.8 Å (Fig. 1c). Across the matched pairs, MPA reproduces the DFT adsorption energies with a slope near unity (*m* = 1.04, *R*^2^ = 0.98, *n* = 11) and a MAE of 3.6 kcal mol^−1^, decomposing as 3.3 kcal mol^−1^ for molecular (*n* = 7) and 4.1 kcal mol^−1^ for dissociative adsorption (*n* = 4) (Fig. 1d and Fig. S1). Additionally, we find that single-point PBE-D2*(*N*) calculations on the MPA geometries recover the MLP energies to within 1.3 kcal mol^−1^, whereas the MLP-optimised structures lie on average 3.1 kcal mol^−1^ above the DFT minima. The foundation model therefore locates the relevant minima on a well-shaped potential energy surface and closely reproduces their relative ordering, but does not replace DFT in resolving near-degenerate configurations, which is why every retained candidate in this work is re-optimised at the PBE-D2*(*N*) level of theory. The structural agreement at the individual level is illustrated in Fig. 1e, where selected internal coordinates of the deprotonated H_2_S adsorbate in the global minimum between the DFT and MPA datasets differ by an average of 0.01 Å.

### High-throughput adsorption sampling

Candidate adsorption sites were first enumerated geometrically on each termination using Delaunay triangulation of the surface atom centres, restricted to the irreducible cell, yielding between 7 (MgO(100)) and 91 (silica) chemically distinct sites. H_2_S was then placed S-down at each site to generate initial guesses, which were relaxed with the selected foundation MLP, *i.e.*, MPA. The resulting minima were pruned by structural similarity and an adsorption-energy window of 40 kcal mol^−1^ from the global minimum of each surface, re-optimised at the PBE-D2*(*N*) level, and finally subjected to hybrid HSE06 single-point calculations and to vibrational analysis for spectral simulation. Leveraging the demonstrated accuracy of the selected MLP foundation model, we then sampled all adsorption configurations in a high throughput fashion on silica, as well as the pristine and vacancy-bearing MgO(100) surface and the predominant terminations of Mg_2_SiO_4_ at the equilibrium shape at 0 K, *i.e.*, (010), (120), (101), (111), (001) and (021). This broad configurational sampling yielded a chemically diverse range of adsorbate structures, which were categorized based on their adsorption energy and the maximum H–S distance between adsorbate atoms, as shown in Fig. 2.

**Fig. 2:**
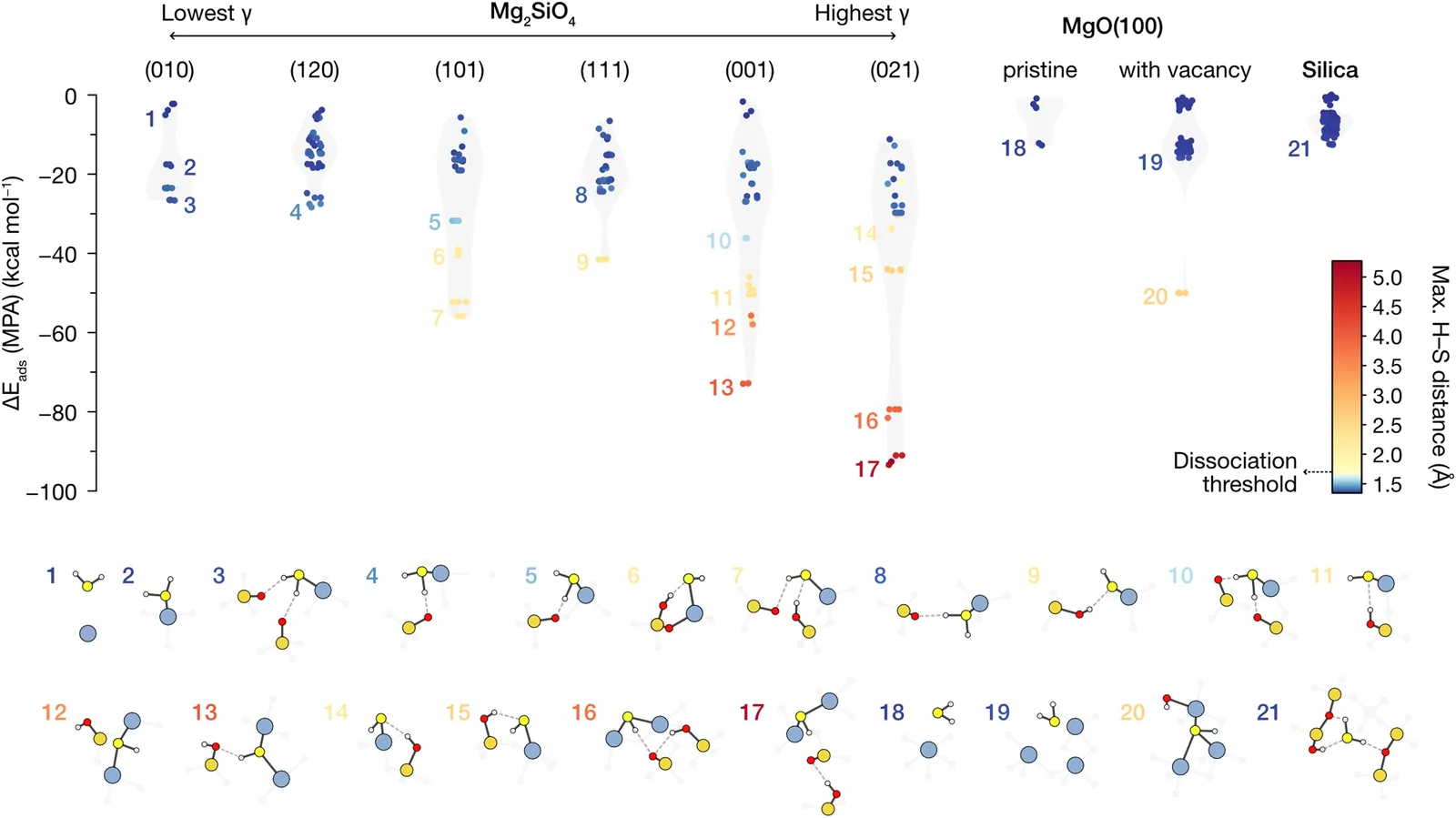
Potential energies of adsorption of H_2_S on the simulated terminations of Mg_2_SiO_4_, pristine and vacancy-bearing MgO, and silica, optimized using the best-performing MLP in the benchmark phase, *i.e.*, MPA. Adsorptions are colour-coded with respect to the maximum H–S distance between adsorbate atoms. Relevant adsorptions are labelled in the jitter plots, with their corresponding structures shown below. Atom colour codes are provided in Fig. 1e.

For Mg_2_SiO_4_, our simulations show that dissociative adsorption is favoured on more unstable, higher surface energy-bearing terminations, while more stable and extended planes such as (010) and (120) only contained both physisorbed and molecularly adsorbed configurations, the most stable forming hydrogen bonds (H-bonds) with neighbouring surface O moieties with adsorption energies of −28.4 kcal mol^−1^. In the case of terminations with relative intermediate surface energy, *i.e.*, (101) and (111), our calculations showed stable surface adsorptions corresponding to dissociatively bound H_2_S, wherein the HS moiety remained bound to a surface Mg atom, whilst the dissociated H transferred to an adjacent surface O atom and remained oriented towards the HS group, forming a highly directional H-bond. They displayed adsorption energies up to around −55.0 kcal mol^−1^ in the most stable configuration. These dissociated adsorption complexes are in good agreement with previous studies on the dissociative adsorption of H_2_O on TiO_2_ ^30,31^ and in the deprotonation of H_2_S on CeO_2_ ^4^ using DFT+*U* calculations. Only on higher surface energy-bearing terminations, such as (001) and (021), did we find dissociative adsorptions, where the resulting H and HS moieties on surface O and Mg, respectively, diffuse to more coordinatively unsaturated sites, characterized by large negative adsorption energies up to −93.4 kcal mol^−1^.

Our MLP-based screening predicted that the presence of Schottky-pair vacancies on MgO(100) introduces distinct reactive environments compared to pristine facets, favouring dissociated H_2_S species with adsorption energies of around −50.0 kcal mol^−1^; in contrast, the results showed that the pristine MgO(100) termination, which is known to interact weakly with probe molecules,^32^ was unable to readily activate and deprotonate H_2_S, in agreement with previous theoretical studies,^7^ only attaining molecular adsorptions on surface Mg sites.

Finally, our high-throughput procedure only predicted physisorbed H_2_S on silica with adsorption energies down to −12.5 kcal mol^−1^, whose adsorptions were dominated by the presence of one or multiple H-bonds to and from surface silanol (SiOH) groups.

### Structural refinement and theoretical simulation of IR spectra

Given the chemical diversity of the obtained adsorbate structures on the surfaces, we refined the geometrical structures by performing a geometry- and energy-based pruning and subsequent optimization at the PBE-D2*(*N*) level of theory, followed by single-point energy calculations using the HSE06 hybrid functional. This was aimed at accurately describing the interatomic distances of relevant atoms using a parametrized dispersion term prior to the determination of spectral features, as well as correctly obtaining the adsorption energies of each configuration on the different surfaces at a higher level of theory. The Gibbs energies of adsorption (see the Methods section) of all systems at 150 K *vs*. the maximum H–S distance are displayed in Fig. 3a. In this, our calculations showed a similar scenario to that in the MLP sampling, *i.e.*, H_2_S chemisorption and dissociation on Mg_2_SiO_4_ occur on higher surface energy terminations and on MgO(100) with NN and NNN Schottky-type defects, while on both silica and pristine MgO(100), H_2_S can only physisorb and molecularly adsorb, respectively, with H-bonding being the major driver of stabilization in the former. We note that the generation of Schottky pair point defects on the MgO(100) surface, which is aimed at emulating the conditions found at the experimental settings (where the existence of steps, kinks and vacancies dominate molecular dissociation^33,34^), is favoured by 12.1 kcal mol^−1^ on MgO(100) with NN Schottky defects, as opposed to NNN defects, which is in line with previous works;^35^ thus, we envision the local environment of the experimental surface to be more accurately represented by a MgO(100) model surface with NN Schottky-type defects.

**Fig. 3:**
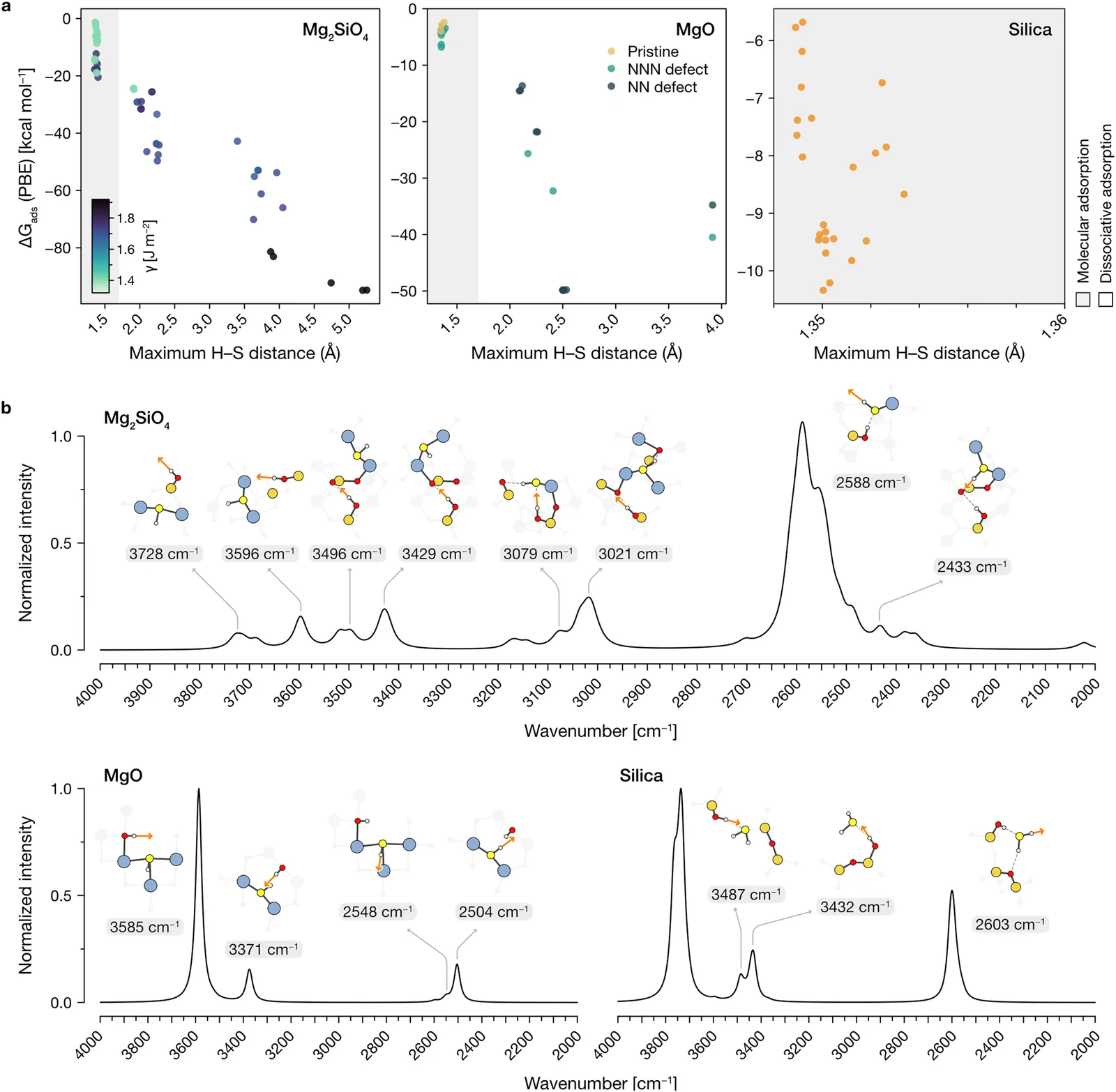
(a) Scatter plots of H_2_S adsorption on Mg_2_SiO_4_ (left hand panel), MgO (central panel) and amorphous silica (right hand panel), depicted as Gibbs energy of adsorption *vs*. maximum H–S distance among the H_2_S atoms. In this representation, the points lying inside the shadowed areas (with a maximum H–S distance below 1.7 Å) depict molecular adsorptions, while the points outside these areas correspond to dissociative adsorptions with diffusion of the resulting H and HS moieties on the surface. The points in the Mg_2_SiO_4_ and MgO plots are coloured with respect to the surface termination where the corresponding adsorptions occur: for the former, adsorptions are colour coded with the surface energy of their termination. The points in the MgO plot are coloured by surface type: pristine MgO(100) (tan) and surfaces containing NN or NNN Schottky-type defects (dark and light blue, respectively). (b) DFT-simulated compound IR spectra of Mg_2_SiO_4_ (topmost panel), MgO with a single Schottky-pair vacancy (bottom left-hand panel) and hydroxylated amorphous silica (bottom right-hand panel). Vibrational modes of relevant peaks are included as insets, depicted as top-view atomic representations of the local environment of the H_2_S adsorbate qualitatively showing the displacements along each feature as orange arrows. Atom colour codes are presented in Fig. 1e.

In view of the obtained results, we then sought to elucidate the vibrational features of H_2_S adsorbed on silica, as well as on the surfaces of Mg_2_SiO_4_ and vacancy-bearing MgO. To this end, multiconfigurational IR spectra were simulated at the DFT level for the three prototypical systems (Fig. 3b) by calculating the vibrational frequencies in the harmonic approximation and Born effective charge tensors for the relevant localized modes (see the Methods section). For Mg_2_SiO_4_, this yielded a multitude of O–H stretching bands ranging from 3728 to 3429 cm^−1^, corresponding to modes of dissociated adsorptions where the newly formed OH moiety faces surrounding surface species at various degrees, with the most prominent red shift corresponding to H-bonding with surface O moieties. In addition, more prominent O–H stretching modes were predicted to appear at lower frequencies such as 3079 or 3021 cm^−1^, which correspond to highly directional H-bonding between a dissociated H transferred on a surface O atom and the parent deprotonated HS species, or to nearby undercoordinated surface O sites. While these configurations are theoretically stable, we highlight the significant strain associated with these highly energetic modes. We expect these modes to be quenched at operating temperatures due to thermally induced atomic rearrangement. Finally, calculations show that a broad peak appears around 2588 cm^−1^, corresponding to the H–S stretching mode, with a long tail including peaks arising from a variety of configurations with different orientations of the HS adsorbate, which also has the ability to undergo H-bonding with surface species.

For MgO(100) with a nearest-neighbour Schottky pair point defect, our calculations showed that significant recombination of the surface atoms around the divacancy upon H_2_S dissociative adsorption yields structures with marked directional H-bonding to surface hydroxyl groups at the opposite site of the vacancy, characterized by strongly red shifted features such as the O–H stretching mode at 3585 cm^−1^ and the H–S stretching peak at 2504 cm^−1^, highlighting the ability of defect-bearing MgO(100) to strongly dissociate H_2_S.

Finally, for silica, calculations showed the emergence of red shifted O–H stretching modes at frequencies of around 3487 to 3432 cm^−1^, corresponding to H-bonded silanol species interacting with physisorbed H_2_S, alongside the common O–H stretching band at around 3750 cm^−1^. Furthermore, simulations predicted the existence of a red shifted H–S stretching band compared to the gas-phase at 2603 cm^−1^, corresponding to H_2_S stabilized through H-bonding with surface hydroxyl groups.

### Comparison between DFT-simulated and experimental IR spectra

To test the predicted surface interactions against experimental data, *in situ* IR spectra of H_2_S on amorphous silica, Mg_2_SiO_4_ and MgO were obtained (Fig. 4). The experimental setup allowed for the monitoring of the spectral evolution across the chemically diverse oxide surface under controlled atmosphere and temperature conditions.

**Fig. 4:**
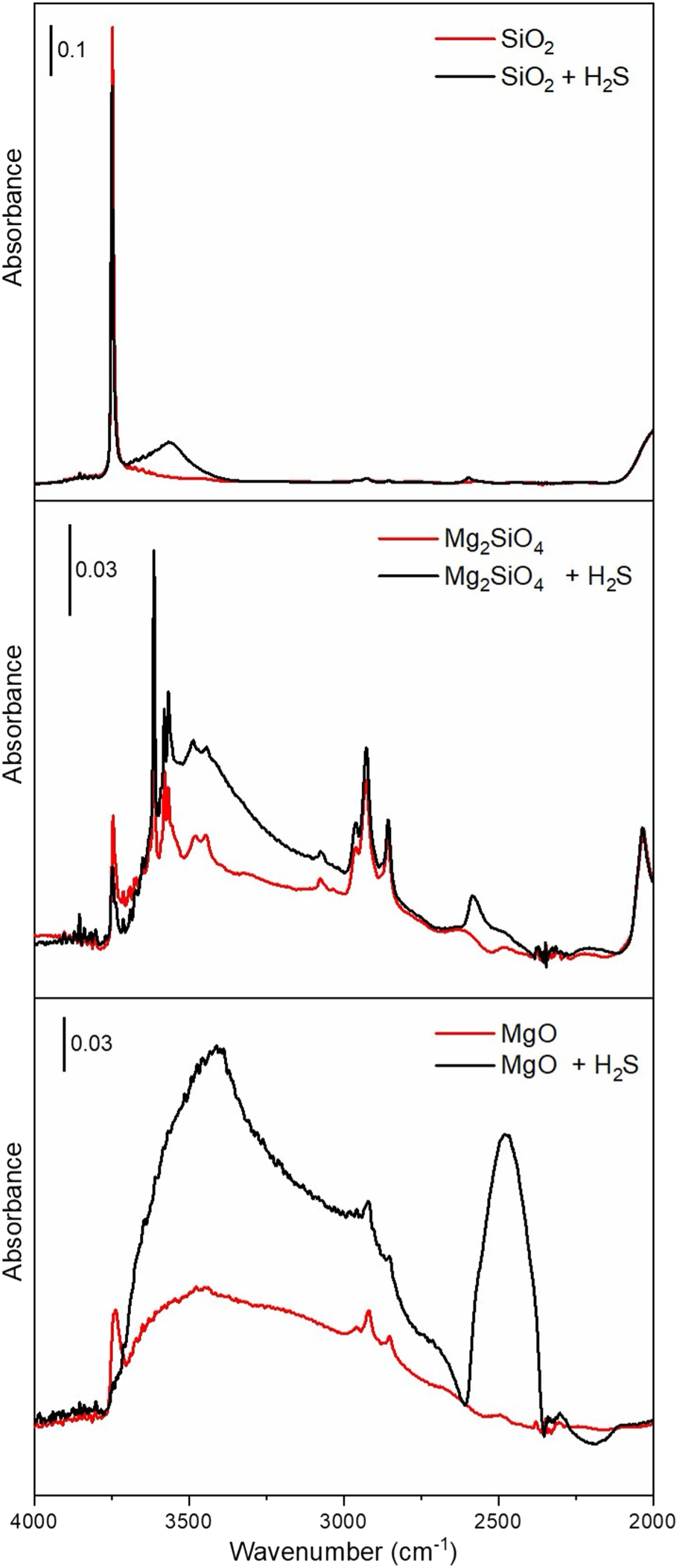
Experimental IR spectra of SiO_2_, Mg_2_SiO_4_, and MgO immediately after activation (red curves) and at maximum H_2_S coverage (black curves) at 150 K.

We first turn to the high-frequency O–H stretching region, where our computational framework successfully captures the emergence of O–H stretching modes arising from intermolecular H-bonding. In the silica spectra, we observe that a fraction of the isolated surface hydroxyl groups, associated with a peak at 3747 cm^−1^ in the activated sample,^36^ interacts with H_2_S, giving rise to a broad band centred around 3560 cm^−1^. Its position is compatible with our computational predictions, although slightly less redshifted; this discrepancy is inherently linked to the diversity and complexity of the experimental surface, in contrast to our single-structure amorphous silica model. For Mg_2_SiO_4_, the simulated spectra successfully reproduce the experimentally observed increase in spectral features below 3700 cm^−1^, which is assigned to the stretching of H-bonded surface hydroxyls generated immediately upon H_2_S dissociation. Similarly, for the defect-bearing MgO(100) surface, our calculations correctly predict a massive red-shifting of the O–H stretching frequencies and the emergence of distinct low-frequency modes, confirming that the hydroxyls formed upon dissociation undergo strong H-bonding around the defective site.

The structural fidelity of our models is most prominently validated in the characteristic H–S stretching region. Fig. 5 shows a direct comparison of the experimental and computed spectra. For silica, the theoretical compound spectrum provides an accurate prediction of both the exact band shape and the location of the H–S stretch at around 2600 cm^−1^. Its position is also in agreement with previous experimental studies.^37^

**Fig. 5:**
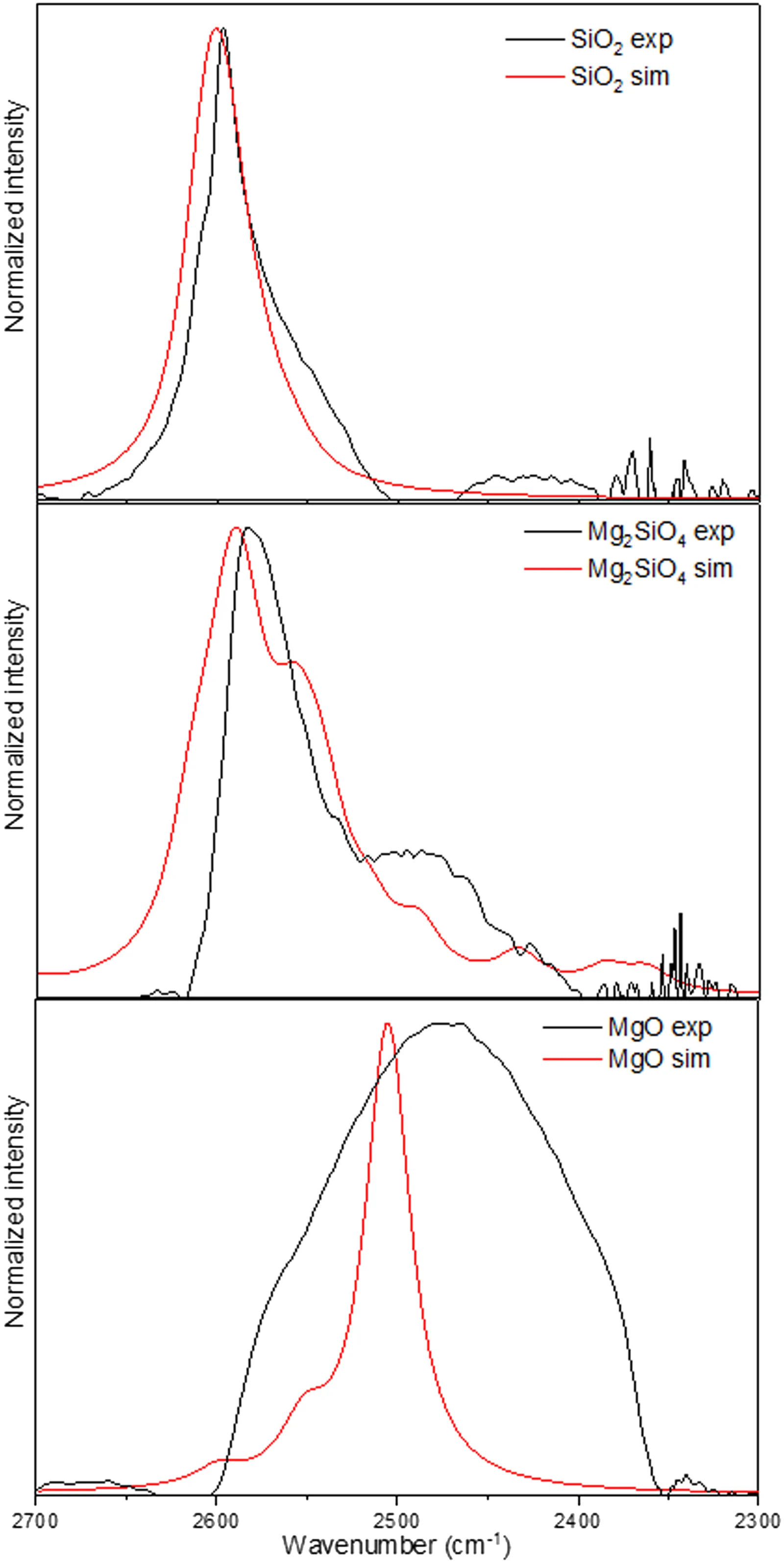
Comparison between baseline-subtracted normalized experimental (black lines) and DFT-simulated (red lines) IR spectra in the H–S stretching region of H_2_S adsorption on silica, Mg_2_SiO_4_ and MgO.

For Mg_2_SiO_4_, the maximum of the experimental spectrum is redshifted to 2578 cm^−1^ with a broad low-frequency tail, which is reproduced by our simulations. As illustrated by the vibrational mode analysis in Fig. 3b, this extended tail is not a single feature, but rather a convolution of multiple less energetic H–S stretching modes belonging to dissociated surface SH moieties, whose bonds have been significantly weakened due to strong, highly directional H-bonding with surrounding surface O sites.

Finally, the MgO system exhibits the most profound spectral shifts with a maximum of the experimental signal at 2470 cm^−1^. Both theory and experiment demonstrate that the H–S stretching mode on MgO is substantially red-shifted compared to that on silica and Mg_2_SiO_4_. This reflects a highly activated bond, indicative of the intense polarizing field and the strong dissociative character of the Schottky divacancy pairs. We note that the simulated H–S peak for MgO is narrower than the experimental broad band; this is an expected limitation, as our theoretical dataset for MgO is currently limited to a single crystallographic facet featuring only one type of divacancy, thereby capturing a less diverse distribution of stable minima than the complex experimental surface, which may also exhibit highly reactive low-coordination Mg ions at kinks or corners. Thus, the DFT spectra computed for the MLP-screened minima correctly predict the general trend in the location of the H–S stretch peak maximum across the three materials: silica (2603 cm^−1^) > Mg_2_SiO_4_ (2588 cm^−1^) > MgO (2504 cm^−1^). This progressive frequency shift serves as a direct, observable descriptor for the strength of the surface interaction.

## Methods

### Experimental details

#### Materials

Forsterite (Mg_2_SiO_4_) was obtained by heating in air, at 1073 K for 24 h, an amorphous magnesium silicate previously synthesized by Yamamoto *et al.* through the evaporation of Mg(OH)_2_ and SiO_2_ precursors in a high-temperature plasma (∼10 000 K).^38^

Pyrogenic amorphous silica (SiO_2_, Aerosil OX 300) was purchased from Evonik (formerly Degussa).

Magnesium oxide (MgO) was synthesized *in situ* in the IR cell (*vide infra*) from the precursor Mg(OH)_2_ by stepwise thermal decomposition under vacuum. First, Mg(OH)_2_ was heated at 393 K overnight. Subsequently, the material was outgassed at increasing temperatures (523, 593, 693 and 1073 K) until reaching a residual pressure of 1 × 10^−3^ mbar. Then, the material was exposed twice to O_2_ for 20 minutes with an intermediate outgassing step. Finally, the material was outgassed and cooled down to room temperature.

#### FT-IR spectroscopy

The samples were pressed in the form of self-supporting pellets, appropriate for IR measurements in transmission mode, and placed in a quartz low-temperature infrared cell equipped with KBr windows, which can be cooled using liquid N_2_. This cell was directly connected to a conventional vacuum line, enabling all thermal treatments and adsorption–desorption experiments to be performed *in situ*. Prior to the measurements, the pellets were activated to remove undesired adsorbed molecules from the material surfaces, primarily atmospheric H_2_O. Specifically, forsterite was outgassed at 673 K for 1 hour, silica at 973 K for 2 hours, and MgO was immediately investigated after the *in situ* synthesis. H_2_S adsorption was monitored using a Bruker Equinox 55 spectrometer equipped with a DTGS detector, operating at a resolution of 4 cm^−1^, averaging 128 scans to achieve an adequate signal-to-noise ratio.

### Computational details

#### Design of active surfaces

All periodic density functional theory (DFT) calculations were performed using the Perdew–Burke–Ernzerhof (PBE) functional^39^ and the projector augmented-wave (PAW) approximation^40^ as implemented in the Vienna *Ab Initio* Simulation Package (VASP, version 6.4.3).^41^ In all calculations, an energy cutoff of 500 eV was considered to construct the plane-wave basis set. A *k*-point density of ∼21 *k*-points per Å^−1^ (approximating to the closest even number in each axis) was used to sample the reciprocal space along all axes using a Γ-centred *k*-point grid, following successful convergence in a previous work on Mg_2_SiO_4_.^20^ Bulk structures of Mg_2_SiO_4_ and MgO were obtained from the Materials Project database,^42^ and their lattice constants were optimized by plotting the cell volume against its energy using the Birch–Murnaghan equation of state, with the original lattice parameter varied from 98 to 102%. All structures were optimized using a conjugate gradient iteration scheme with a force-based convergence criterion of 0.015 eV Å^−1^. In all instances, long-range interactions were computed using Grimme's D2 dispersion model^43^ for all atoms, including an empirical correction for Mg cations as developed in the D2*(*N*) model to account for its highly ionic character and much lower polarizability than atomic magnesium.^44^

Given that calculations are performed using periodic boundary conditions, the choice of surfaces, either multi-faceted, defective or amorphous, was made based on the existence of local environments that more accurately depict the sites present in the experimentally produced nanoparticles. Slab models for MgO(100) (belonging to the *Fm*3̄*m* space group) and the six facets present in the equilibrium shape of Mg_2_SiO_4_ at 0 K, *i.e.*, (120), (101), (001), (111), (021) and (010) (in the *Pbnm* space group), were constructed with the in-house tool *polycleaver*.^24^ Single Schottky defects were introduced on MgO(100) slabs by removing one surface Mg^2+^ and one surface O^2−^ ion with two geometric variants: (i) nearest-neighbour (NN), where vacancies are adjacent, and (ii) next-nearest-neighbour (NNN), where vacancies are separated by one lattice site. To maintain slab non-polarity and avoid unphysical dipole moments, identical Schottky defect pairs were placed on both symmetric sides of the slab for all configurations. A hydroxylated slab of amorphous silica was obtained from a previous work.^45^ Atomic representations of all modelled slabs are displayed in Fig. S2.

#### Design of initial guesses for adsorption sampling

An autonomous, fully machine-based and chemically guided algorithm was used to thoroughly assess the configurational space of the adsorption of H_2_S on all surfaces. The following procedure avoids human intervention based on intuition avoiding any bias in the surface exploration procedure. First, H_2_S was positioned S-down on each surface site obtained through a Delaunay triangulation from the atom centres of all surface sites, which are defined as atoms whose centre is not eclipsed by any other species’ covalent radii along the *c* axis. In this scheme, centres, vertices and midpoints of the triangles roughly depict hollow, atop and bridge sites on the surface.^24,25,46^ To avoid multiplicity of sampled sites, only sites within the irreducible unit cell were considered, whilst the remaining were discarded; additionally, points within 0.7 Å of each other were deemed equivalent and only one of them was considered. This yielded chemically distinct sites, ranging from 7 sites on MgO(100) to 91 on amorphous silica, to accurately sample all potential surface adsorption sites. The adsorbate sequentially approached the surface until its ionic radii overlapped, at which point the adsorbate atoms’ *z* coordinate was increased by 1 Å to avoid falling into spurious energetic pitfalls.

#### Dataset generation and MLP benchmark

All reference adsorption geometries were built from periodic optimizations at the PBE-D2*(*N*) level of initial guesses of H_2_S adsorbed on Mg_2_SiO_4_(001). These DFT calculations define the reference dataset, comprising a total of 43 geometries and adsorption energies.

To assess the fidelity of MLPs to accelerate our calculations, we then compared the stable adsorbate structures in the reference dataset to those arising from the optimization of the initial guesses through a series of models, namely Meta's Universal Models for Atoms^47^ trained on the Open Catalyst 2020 dataset^48^ (UMA-OC20) and the available foundation models within the MACE architecture, *i.e.*, a model trained on the MatPES dataset both at the PBE and r2SCAN levels^49^ (MatPES-PBE and MatPES-r2SCAN, respectively), a model trained on the Open Materials 2024 dataset^50^ (OMAT24) and the MACE-MPA-0 model trained on the Materials Project Trajectory (MPTrj) and Alexandria datasets (hereafter MPA).^26^ For each model, an individual dataset (model dataset) was constructed.

For a consistent comparison between the reference and model datasets, local adsorption clusters were extracted from each full structure using a chemically informed rule set. Each cluster contained the atoms in the H_2_S adsorbate, as well as the nearest surface Mg atom to S and, in cases where H_2_S dissociation occurs (defined by a maximum H–S distance above 1.5 Å in the optimized structure), the nearest surface O atom to any H. To account for slight divergences in adsorbate geometries, a cluster was also built with the second-nearest surface Mg atom when its Mg–S distance was within 0.3 Å of the shortest distance. Reference and MLP cluster datasets including cluster geometries and the adsorption energies of their respective full-size counterparts were constructed.

Each structure from a given MLP cluster dataset was compared against all clusters with the same protonation state (protonated or deprotonated) in the reference cluster dataset. For every DFT–MLP cluster pair, the root-mean-square deviation (RMSD) was computed using a brute-force order matcher implemented in the pymatgen library, which superimposes the two clusters over all rigid rotations, translations and atom-order permutations, considering the minimum-image convention under periodic boundary conditions. In this scheme, a cluster pair was considered a match if their RMSD was below 0.4 Å, which we found to balance geometric tolerance with chemical fidelity.

Because adsorption optimizations can yield duplicate or quasi-identical minima, we implemented a multi-stage pruning to ensure a one-to-one correspondence between unique adsorption motifs. First, we performed an intra-dataset pruning: when two clusters of identical composition displayed an RMSD below 0.05 Å, only the configuration with the lowest adsorption energy was retained. This procedure prevents over-counting of symmetry-equivalent or trivially rotated sites. At the matching stage, we observed that multiple MLP clusters can match the same DFT cluster. Two matching rules are used; to assess whether a model's dataset contains a given reference structure (Fig. 1a–c), each reference structure takes its lowest-RMSD partner among the MLP clusters of the same protonation state, without a one-to-one constraint; one MLP structure may therefore account for more than one reference structure. Subsequently, to compare adsorption energies (Fig. 1d), matched pairs are required, so only the lowest-RMSD pairing is retained for each MLP structure, leaving 11 pairs for MPA. After pruning, each surviving DFT–MLP cluster pair defines a unique data point with DFT and MLP adsorption energies [Δ*E*_ads_ (DFT) and Δ*E*_ads_ (MLP), respectively], and a structural RMSD. These adsorption energies were calculated as follows: Δ*E*_ads_ = *E*_complex_ − *E*_surface_ − *E*_H_2_S_where *E*_complex_ is the calculated potential energy of the surface-H_2_S complex, *E*_surface_ is the potential energy of the bare surface, and *E*_H_2_S_ the energy of the H_2_S molecule in the gas phase. Direct energetic and geometric comparisons between matching pairs in the reference and MLP cluster datasets are shown in Fig. S1 and S3–7, respectively. Subsequent analyses were conducted by selecting the most stable adsorbate candidates on each surface, within 40 kcal mol^−1^ of its global minimum; this was aimed at capturing a wide range of chemically diverse adsorbates, whilst limiting the computational cost. As aforementioned, these structures were subsequently refined by optimizing at the PBE-D2*(*N*) level.

#### Calculation of thermodynamic properties and Gibbs energies of adsorption

To determine the thermodynamic stability of the various adsorption configurations at the experimental temperature of 150 K, Gibbs energies of adsorption (Δ*G*_ads_) were computed using a harmonic oscillator approximation for the vibrational degrees of freedom. The total Gibbs energy for each surface-adsorbate complex (*G*_complex_) and the gas-phase H_2_S molecule (*G*_H_2_S_) was calculated as follows: *G*(*T*) = *E*_pot_ + Δ*G*_corr_(*T*)where *E*_pot_ represents the electronic potential energy obtained from periodic DFT optimizations and Δ*G*_corr_(*T*) is the thermal correction to the Gibbs energy, which was derived using the thermochemistry module included in the Atomic Simulation Environment (ASE).^51^ To mitigate overcorrection from bulk-like spectator modes, particularly in the amorphous silica models, a projected vibrational analysis was employed. This projection considered modes involving the H_2_S atoms and their immediate coordination environment: for Mg_2_SiO_4_ and MgO, the adsorbate atoms and the closest surface Mg site to S were included; for dissociative configurations, the closest surface O site to the deprotonated H was also incorporated. In the case of silica, vibrational modes were projected onto the adsorbate atoms and any surface O or OH moieties within the immediate interaction radius.

To ensure numerical reliability, a quasi-harmonic treatment of low-frequency modes was implemented,^52^ wherein real and imaginary modes with magnitudes below 50 and 100 cm^−1^, respectively, were clamped at a constant value of 50 cm^−1^ before calculating the partition function; imaginary modes were verified to correspond to soft translational diffusion of the rigid H_2_S moiety on the surface. In addition, configurations displaying imaginary frequencies larger than 100 cm^−1^ were not considered true minima and were excluded from the MgO and silica datasets used for spectral analysis; three such weakly bound configurations on the (010) and (120) forsterite facets were retained, since removing them changes the normalised compound spectrum by less than 3%. The final Gibbs energy of adsorption was then defined as: Δ*G*_ads_(*T*) = *G*_complex_(*T*) − *E*_surface_ − *G*_H_2_S(g)_(*T*).

#### Simulation of multiconfigurational IR spectra

Vibrational frequencies and normal modes for the identified stable adsorption configurations were computed by determining their Born effective charge tensors (Z*), required for the calculation of IR intensities, using density functional perturbation theory (DFPT) in VASP. For Mg_2_SiO_4_ and MgO, we only calculated phonons belonging to modes including H_2_S adsorbate atoms and the closest surface Mg site to S, also including the closest surface O site to the adsorbate H in dissociative adsorptions; for silica, atoms of the adsorbate and any surface O or OH moieties were considered. Theoretical IR intensities (*I*_*n*_) for each vibrational mode *n* were derived from the square of the change in the macroscopic dipole moment vector (*μ*) with respect to the mass-weighted normal mode coordinate (*Q*_*n*_). This was computed in VASP *via* the contraction of the atomic Born effective charge tensors with the atomic displacement vectors (*e*_*i*,*n*_):
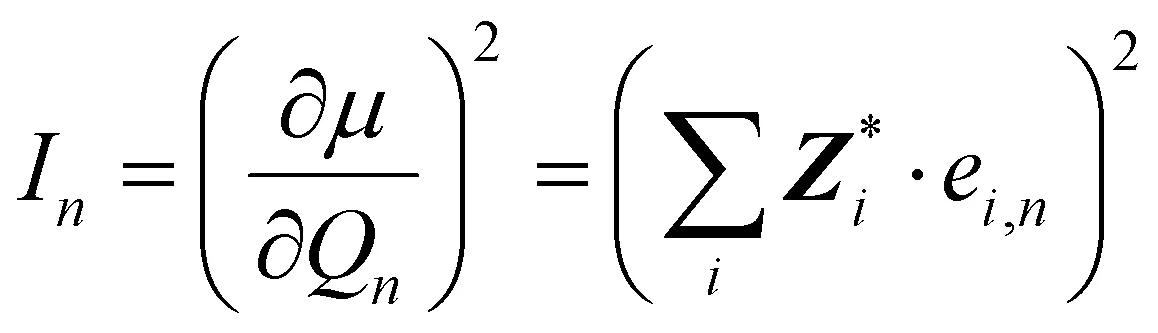
where the sum runs over all considered atoms *i*. The resulting peaks were broadened using a Lorentzian function with a half-width at half-maximum (HWHM) of 15 cm^−1^ to account for broadening effects at the experimental temperature. To account for systematic errors of the functional and for anharmonicity, harmonic wavenumbers were divided by 1.017, the ratio between the largest H–S stretching wavenumber of gas-phase hydrogen sulphide calculated with PBE and the tabulated symmetric H–S stretching peak.

The compound spectrum of each material was constructed as the equally weighted sum of the individual configuration spectra, 
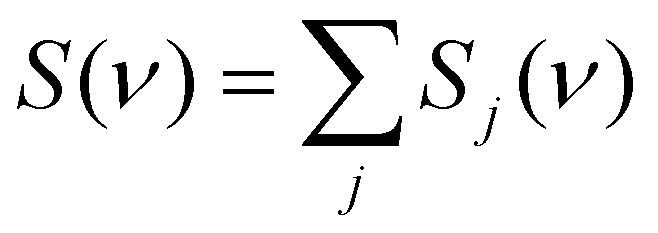
, which was then normalised to its own maximum for comparison with the correspondingly normalised experimental spectra. Individual spectra were not normalised prior to summation, so each configuration contributes in proportion to the intrinsic IR activity of its own modes, in accordance with previous work.^20^

## Conclusions

Understanding chemical adsorption and bond activation on complex oxides remains a key challenge in surface science. While an accurate atomistic description of these interfaces is essential for advancing industrial catalysis, mitigating the effects of pollutants, and deciphering the potential catalytic nature of silicate-based interstellar grains, the vast configurational space associated with chemically diverse reactive sites makes exhaustive sampling impractical using conventional first-principles methods. To overcome these limitations, we demonstrated the key utility of integrating automated and unbiased high-throughput adsorption sampling with readily available state-of-the-art atomistic foundation models. In the present case, this has enabled the unconstrained exploration of H_2_S adsorption and dissociation across a diverse set of prototypical refractory oxide surfaces with wide applications: amorphous silica, Mg_2_SiO_4_ and MgO, the latter in both pristine and defective states.

Importantly, our approach accurately predicted the fundamentally different nature of the interactions that these interfaces exhibit with adsorbates. The MLP-driven thermodynamic mapping successfully showed a variety of surface phenomena, ranging from non-covalent, hydrogen-bonded physisorption on hydroxylated silica and molecular adsorption on pristine MgO and the more stable Mg_2_SiO_4_ surfaces, to dissociative adsorption on defective MgO and the less stable Mg_2_SiO_4_ surfaces.

The structural and geometric fidelity of this high-throughput theoretical framework was assessed against *in situ* IR spectroscopy, with good agreement. By simulating multiconfigurational compound spectra at the DFT level for the MLP-discovered, DFT-refined minima, our theoretical predictions successfully matched the macroscopic experimental observables. The models qualitatively captured band shapes, complex low-frequency tails, and the progressive red-shifting of the characteristic H–S and O–H stretching modes, indicating that the simulated motif ensemble captures the dominant surface species. From an industrial perspective, this combined theoretical and experimental workflow serves as a powerful baseline for studying catalytic systems characterized by complex, heterogeneous surface motifs, paving the way for the rational design of catalysts for energy applications and green chemistry, such as the direct decomposition of toxic H_2_S into valuable H_2_ gas, thereby turning an industrial pollutant into a vital resource for decarbonization strategies. Concurrently, in an astrochemical context, a detailed atomistic picture of H_2_S dissociation on interstellar dust analogues provides critical mechanistic insights into the long-standing problem of sulphur depletion in the interstellar medium, while establishing a reliable atomistic methodology for investigating the surface-catalysed synthesis of prebiotic molecules vital to understanding abiotic chemical evolution in primitive Earth, as a crucial step that ultimately led to the origins of life.

## Author contributions

E. M.-T.: conceptualization, methodology, formal analysis, investigation (theoretical), visualization, and writing – original draft. L. M.: conceptualization, investigation (experimental), formal analysis, and writing – original draft. G. E.-C.: investigation (experimental) and writing – review & editing. N. B.: writing – review & editing. P. U.: conceptualization, funding acquisition, and writing – review & editing. A. R.: conceptualization, funding acquisition, and writing – review & editing. All authors have given approval to the final version of the manuscript.

## Conflicts of interest

There are no conflicts to declare.

## Supplementary Material

NR-OLF-D6NR02993K-s001

## Data Availability

Additional figures supporting this article have been included as part of the supplementary information (SI). Input and output files for all calculations reported in this work, together with the scripts used to build, screen and analyse the adsorption datasets, are openly available from Zenodo at https://doi.org/10.5281/zenodo.19892990. Supplementary information: additional computational details and supporting Fig. S1–S7. See DOI: https://doi.org/10.1039/d6nr02993k.
