# Supplementary material for: Fate of hydrogen sulphide on refractory oxide nanoparticles via foundation model-accelerated atomistic modelling and in situ spectroscopy
Source: Nanoscale. 2026 Nov 2. Online ahead of print. doi: 10.1039/d6nr02993k (PMC13738350; doi:10.1039/d6nr02993k)
Supplement: Supplementary file 1 [file NR-OLF-D6NR02993K-s001.pdf]

Supplementary Information (SI) for Nanoscale.
This journal is © The Royal Society of Chemistry

Supplementary Information

Fate of hydrogen sulphide on refractory oxide nanoparticles via foundation model-accelerated atomistic modelling and in situ spectroscopy

Eric Mates-Torres,[*a] Lorenzo Mino,[*b] Guillermo Escolano-Casado,[b‡] Nadia Balucani,[c] Piero Ugliengo[b] and Albert Rimola[*ad]

a Departament de Química, Universitat Autònoma de Barcelona, 08193 Bellaterra, Catalonia, Spain

b Dipartimento di Chimica and Nanomaterials for Industry and Sustainability (NIS) Centre, Università degli Studi di Torino, via P. Giuria 7, I-10125 Torino, Italy

c Dipartimento di Chimica, Biologia e Biotecnologie, Università degli Studi di Perugia, Via Elce di Sotto 8, I-06123 Perugia, Italy

d Accademia delle Scienze di Torino, Via Maria Vittoria 3, I-10123 Torino, Italy

‡ Present address: Department of Chemistry and Biochemistry, Mendel University in Brno, Zemedelska 1, CZ-613 00 Brno, Czech Republic

** E-mail: eric.mates@uab.cat; lorenzo.mino@unito.it; albert.rimola@uab.cat*

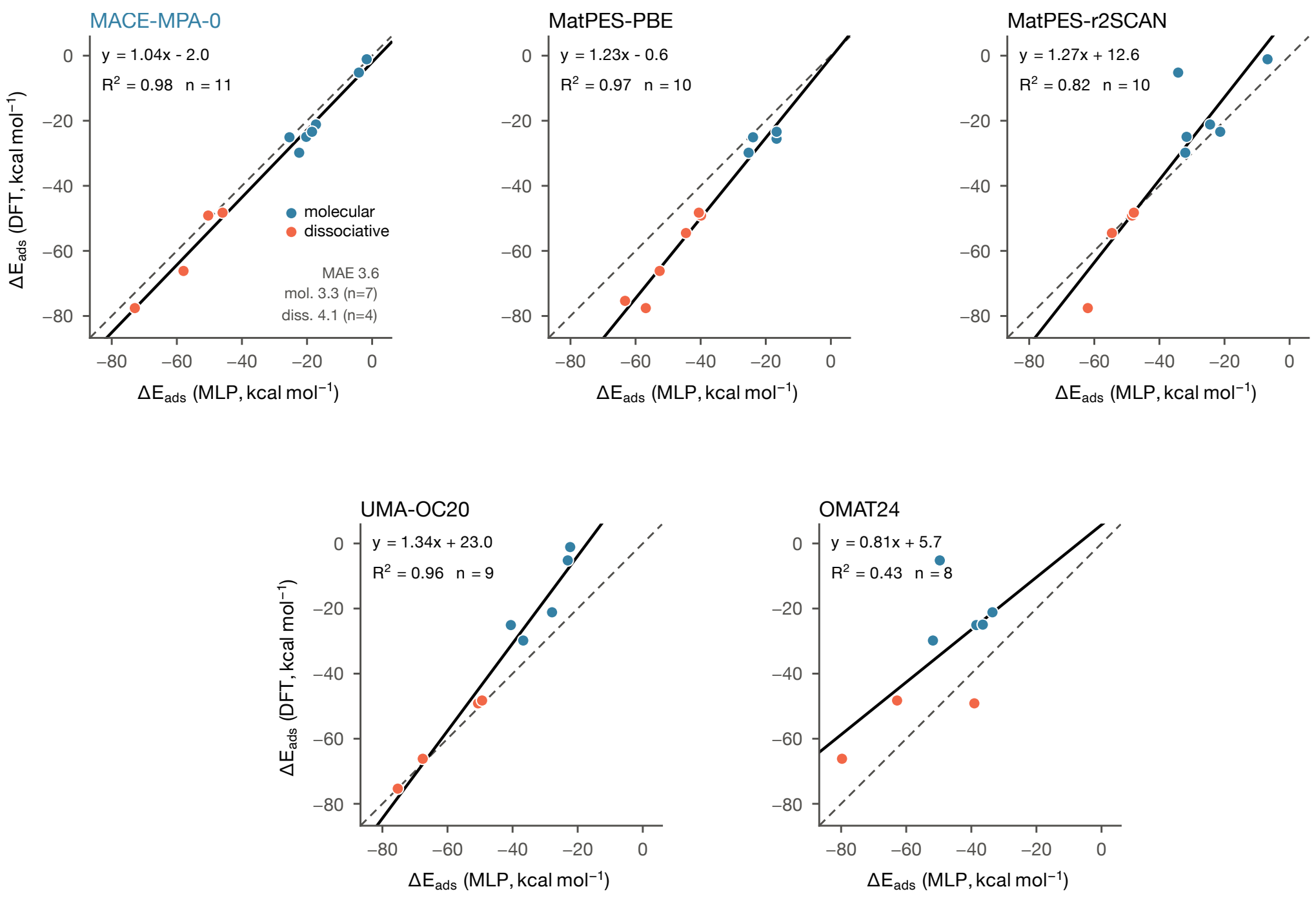

Figure S1. Adsorption energies of RMSD-matched structures between H_2S adsorbate optimizations on Mg_2SiO_4(001) at the PBE-D2*(N) level [ΔE_{ads} (DFT)] and using each machine-learned potential (MLP) [ΔE_{ads} (MLP)]. These include the foundation MLPs included in the MACE architecture: PBE and r2SCAN models based on the MatPES dataset (MatPES), a PBE+*U* model trained on the Open Materials dataset (OMAT), and a PBE+*U* model based on the subsampled version of the Alexandria dataset (sAlex) and the Materials Project Trajectory (MPTrj) dataset (MPA). MACE models were sampled against the oc20 version of of the uma-s-1p1 MLP by the Open Catalyst Project (UMA). In each subplot, a least-square linear regression is calculated, and its R^2 and slope (*m*) are depicted.

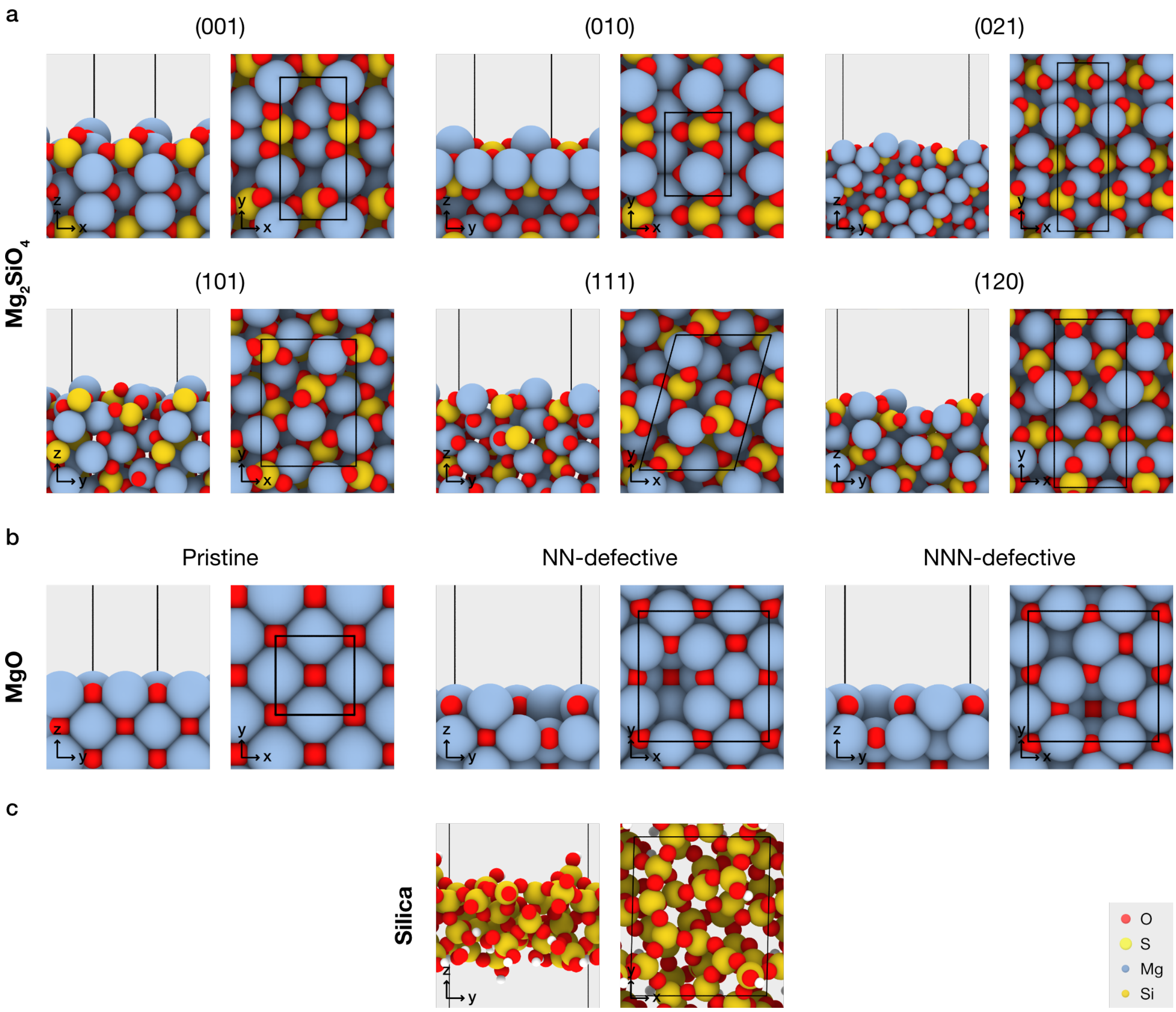

Figure S2. Side and top view atomic representations of the modelled slabs of (**a**) Mg_2SiO_4, (**b**) MgO, and (**c**) amorphous hydroxylated silica. Unit cells are represented with a black outline.

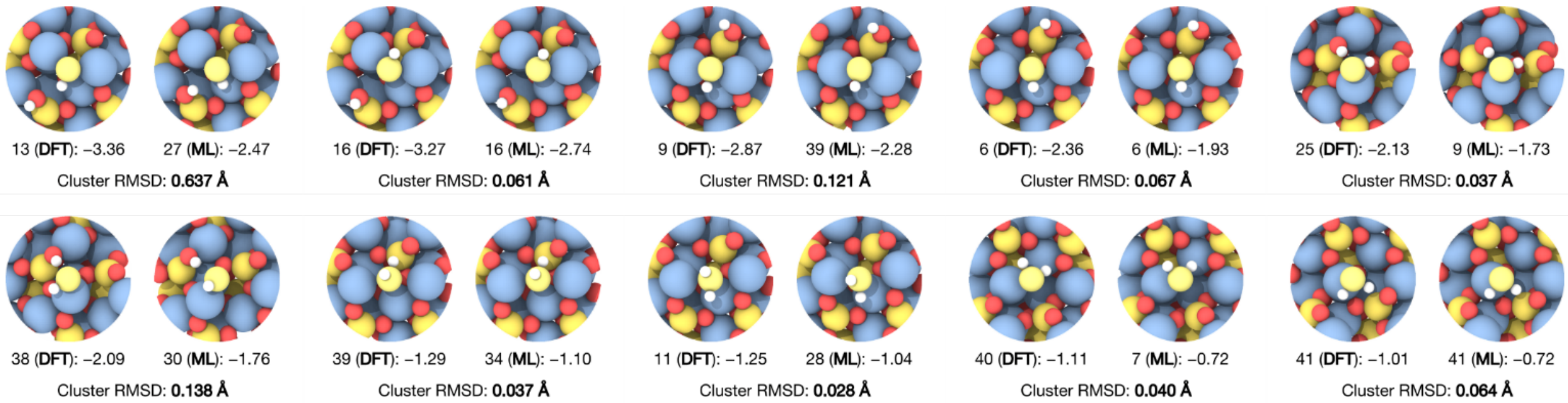

Figure S3. Top view representations of RMSD-matched structures between H_2S adsorbate optimizations on Mg_2SiO_4(001) at the PBE-D2*(*N*) level and using the **MATPES (PBE)** MLP, arranged by DFT adsorption energy. Adsorption energy values are depicted in eV below each structure at the corresponding level of theory. RSMD values are reported under each adsorbate pair.

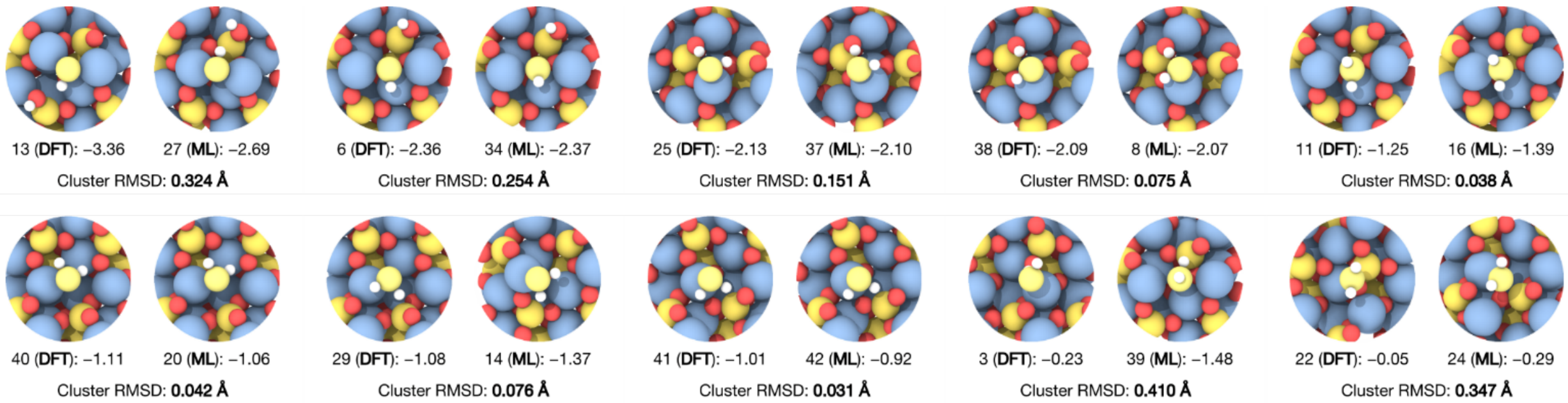

Figure S4. Top view representations of RMSD-matched structures between H_2S adsorbate optimizations on Mg_2SiO_4(001) at the PBE-D2**(N)* level and using the **MATPES (R2SCAN)** MLP, arranged by DFT adsorption energy. Adsorption energy values are depicted in eV below each structure at the corresponding level of theory. RSMD values are reported under each adsorbate pair.

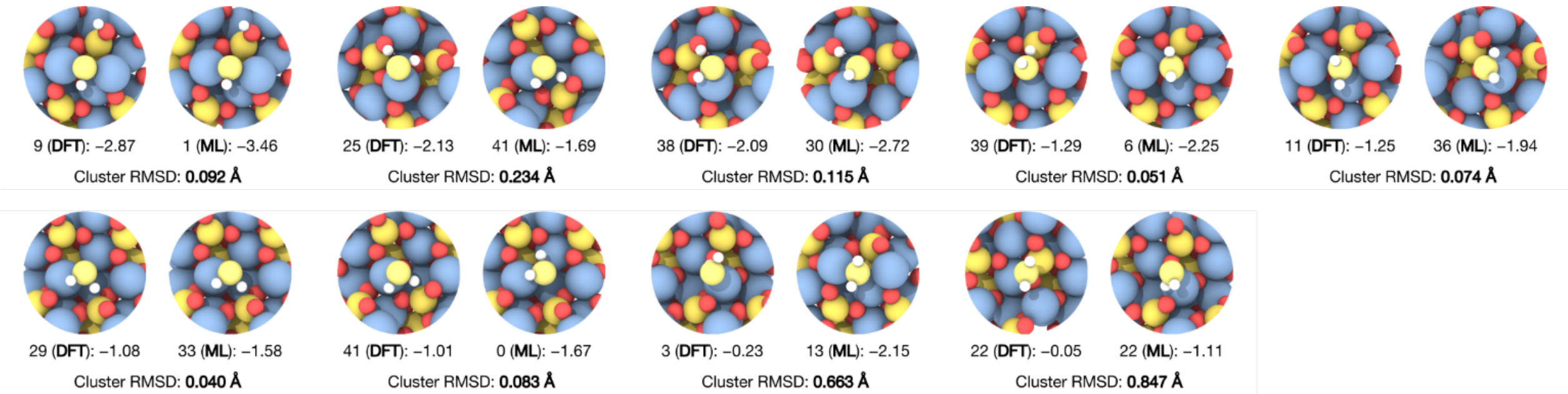

Figure S5. Top view representations of RMSD-matched structures between H_2S adsorbate optimizations on Mg_2SiO_4(001) at the PBE-D2**(N)* level and using the **OMAT** MLP, arranged by DFT adsorption energy. Adsorption energy values are depicted in eV below each structure at the corresponding level of theory. RSMD values are reported under each adsorbate pair.

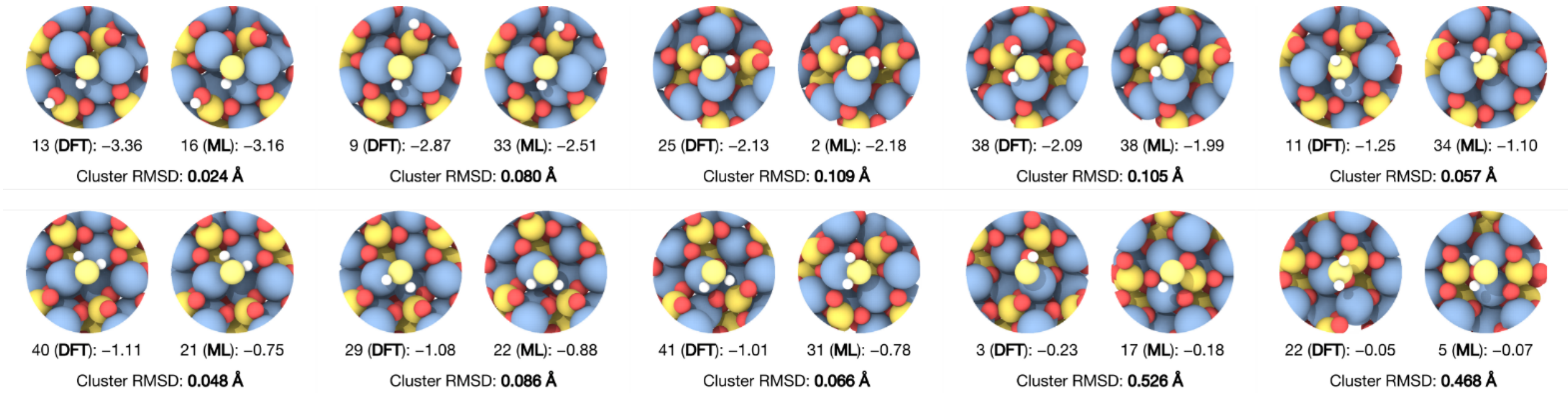

Figure S6. Top view representations of RMSD-matched structures between H_2S adsorbate optimizations on Mg_2SiO_4(001) at the PBE-D2**(N)* level and using the **MACE-MPA** MLP, arranged by DFT adsorption energy. Adsorption energy values are depicted in eV below each structure at the corresponding level of theory. RSMD values are reported under each adsorbate pair.

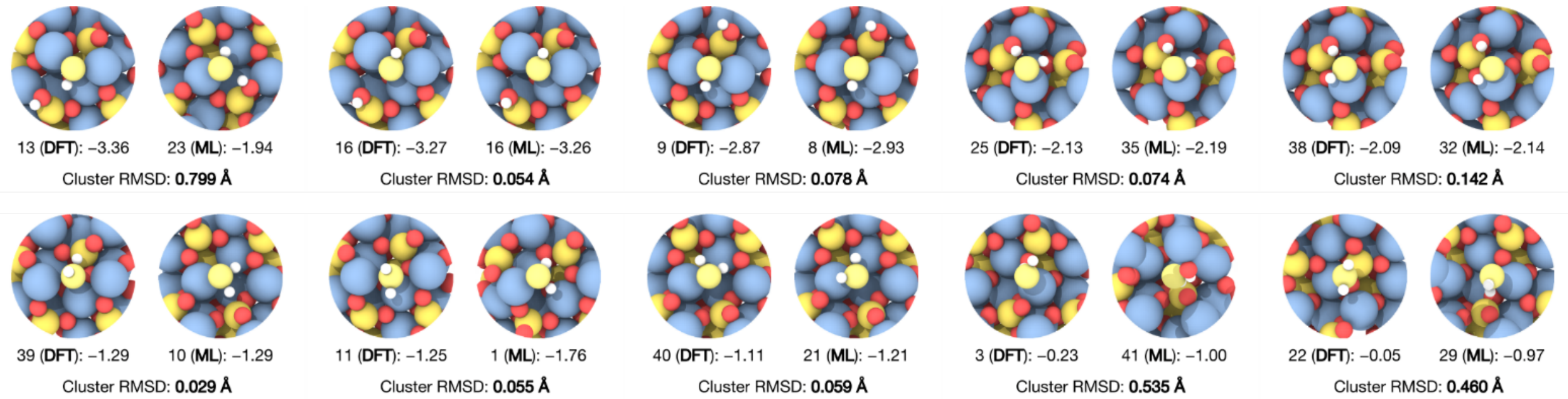

Figure S7. Top view representations of RMSD-matched structures between H_2S adsorbate optimizations on Mg_2SiO_4(001) at the PBE-D2**(N)* level and using the **UMA** MLP, arranged by DFT adsorption energy. Adsorption energy values are depicted in eV below each structure at the corresponding level of theory. RSMD values are reported under each adsorbate pair.
